# Isolated chromoblastomycosis due to *Exophiala bergeri* in an immunosuppressed patient

**DOI:** 10.1016/j.jdcr.2026.09.008

**Published:** 2026-09-10

**Authors:** Therese Murphy, Savannah Alvarado, Mia Do, Sarah Longworth, Alison W. Loren, Emily Y. Chu, Junqian Zhang, Laurel Glaser, Misha Rosenbach

**Affiliations:** a Perelman School of Medicine, University of Pennsylvania, Philadelphia, Pennsylvania; b Department of Dermatology, University of Pennsylvania, Philadelphia, Pennsylvania; c Division of Infectious Disease, University of Pennsylvania, Philadelphia, Pennsylvania; d Division of Hematology-Oncology, Department of Medicine, University of Pennsylvania, Philadelphia, Pennsylvania; e Center for Cell Therapy and Transplant, University of Pennsylvania, Philadelphia, Pennsylvania; f Department of Pathology and Laboratory Medicine, University of Pennsylvania, Philadelphia, Pennsylvania

**Keywords:** Chromoblastomycosis, *E bergeri*, immunosuppression, Medlar bodies, pigmented fungi

## Introduction

Chromoblastomycosis is a chronic, subcutaneous infection caused by pigmented fungi introduced through traumatic inoculation. It occurs most commonly in tropical and subtropical regions, particularly in Latin America, Africa, and Asia, with cases occasionally reported in temperate regions, including North America.^1^ Increased reports of endemic fungal infections outside traditional geographic ranges have been attributed to improved diagnostics, clinical awareness, human migration, and climate change, although empirical evidence specific to chromoblastomycosis remains limited.^2^ Scattered cases have been reported in the United States (US), particularly from Texas and Louisiana.^3^^,^^4^ Immunosuppression may alter the clinical presentation and disease course; thus, atypical lesions in immunosuppressed patients should prompt consideration of uncommon infections, including chromoblastomycosis. Clinically, chromoblastomycosis often begins as a small erythematous macule or papule that can progress into verrucous or plaque-like lesions.^5^ Histologically, it is characterized by Medlar bodies, often described as “copper pennies”.^3^ We present a case of chromoblastomycosis arising in an immunosuppressed patient.

## Case report

A man in his 60s with a history of acute myeloid leukemia (AML), in remission approximately 10 years post allogeneic hematopoietic cell transplant, complicated by cutaneous chronic graft-versus-host disease (cGVHD) that presented as localized plaque morphea, returned to dermatology for follow-up. He had been receiving ruxolitinib 10 mg BID for his cGVHD for 12 months.

He reported an asymptomatic growth on his right ventral forearm present for 1 month. His lifestyle includes yardwork, gardening, tree trimming, and hiking. He wears long sleeves during these activities and did not recall any injury to the area. Upon exam, there was a red, crusted papule roughly 12 mm in size which was slightly boggy on palpation (Fig 1). No other similar lesions were present, and the patient denied any systemic symptoms. Given his immunosuppression, the lesion’s clinical appearance, and history of previous non-melanoma skin cancers, the area was biopsied.

**Fig 1:**
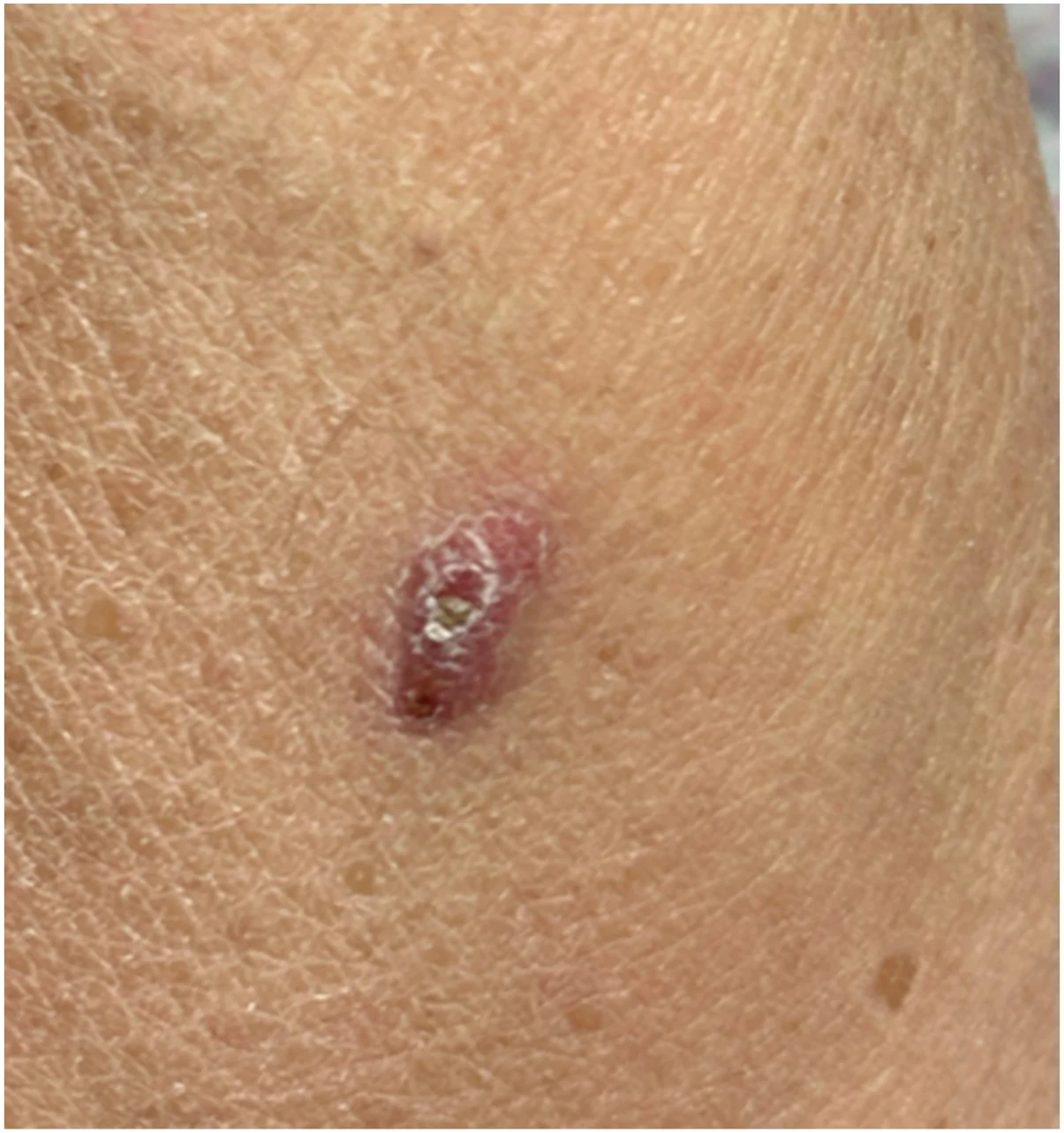
Chromoblastomycosis due to *Exophiala bergeri.* Red, crusted papule approximately 12 mm in diameter on the right ventral forearm.

The biopsy demonstrated mixed granulomatous inflammation in the dermis comprised of histiocytes, multinucleated giant cells, neutrophils, and lymphocytes (Fig 2). Numerous pigmented round fungal bodies resembling “copper pennies” were present within the inflammation (Fig 3). Grocott methenamine silver staining confirmed fungal elements. These findings were most consistent with chromoblastomycosis. Broad-range fungal polymerase chain reaction (PCR) was performed on the biopsy which identified *E. bergeri.*

**Fig 2:**
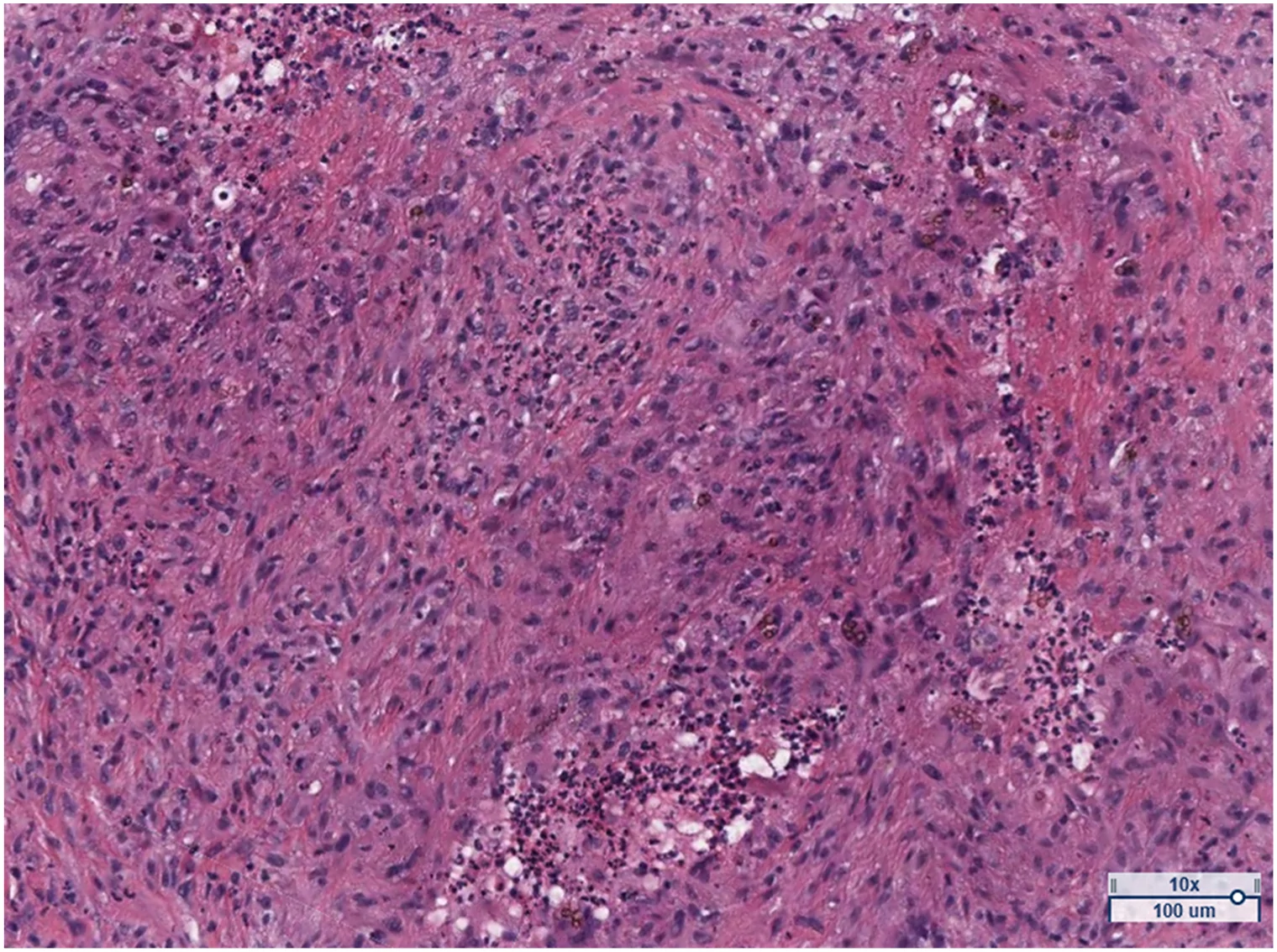
Chromoblastomycosis due to *Exophiala bergeri.* Hematoxylin and eosin stain (10× magnification) demonstrating mixed granulomatous inflammation in the dermis with histiocytes, multinucleated giant cells, neutrophils, and lymphocytes.

**Fig 3:**
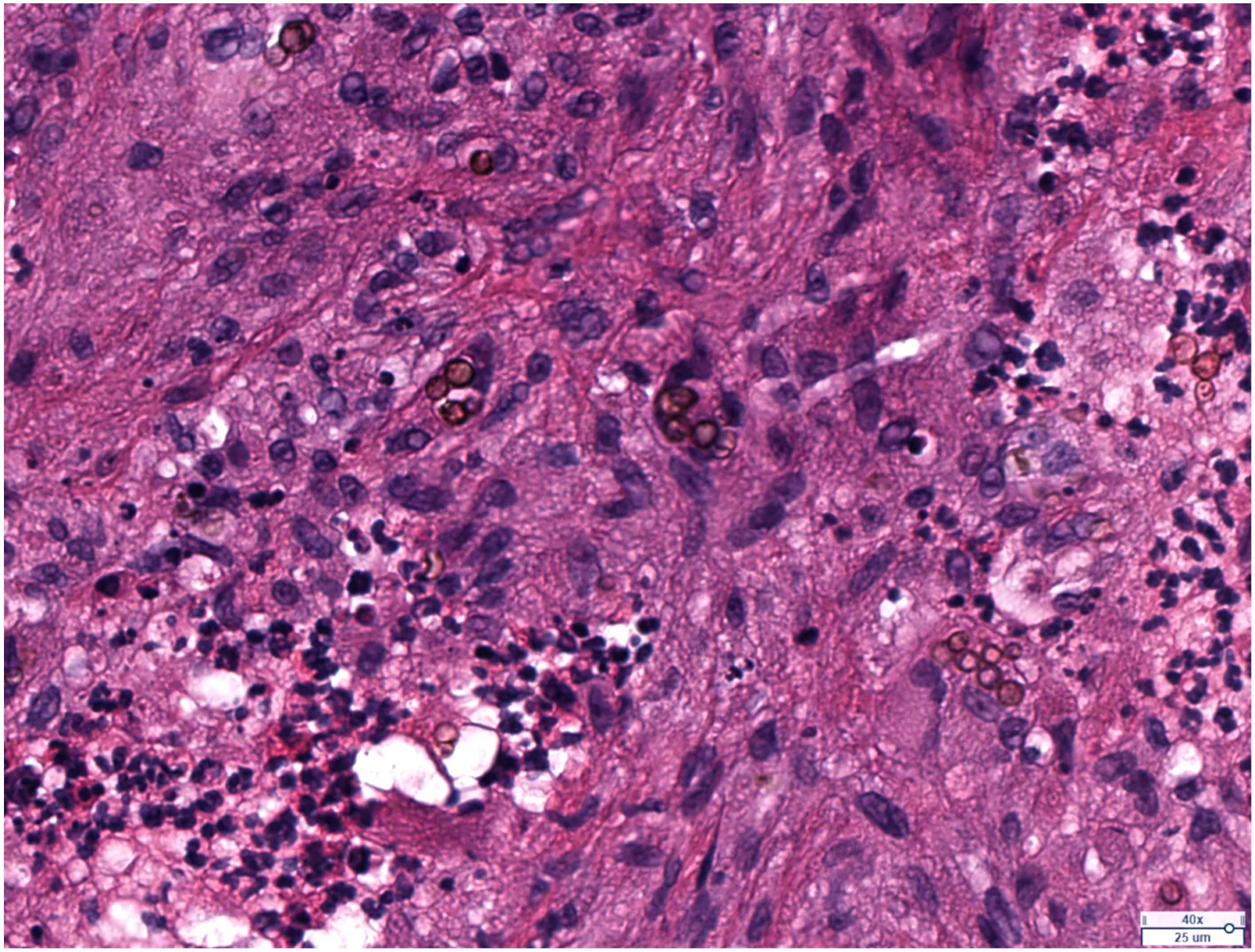
Chromoblastomycosis due to *Exophiala bergeri.* Hematoxylin and eosin stain (40× magnification) demonstrating pigmented muriform cells (Medlar bodies) resembling “copper pennies”.

The patient was referred to infectious disease. Itraconazole 200 mg BID was initiated as first-line therapy, and given the patient’s immunosuppressed status and the localized nature of the lesion, a wide local excision was performed with clear margins. Due to extreme fatigue attributed to itraconazole, and in the setting of complete excision, the medication was discontinued. The patient will continue close clinical follow-up with dermatology and oncology.

## Discussion

Chromoblastomycosis is a pigmented fungal infection of the skin and subcutaneous tissues, most often acquired through traumatic inoculation. Predominantly seen in tropical and subtropical regions, it is associated with agricultural activities such as gardening and farming.^1^ The World Health Organization recognizes it as a neglected tropical disease.^6^ The most common genera worldwide are *Fonsecaea, Cladophialophora,* and *Phialophora*.^1^ *Exophiala* accounts for less than 1% of cases.^1^ To our knowledge, only one prior case of *E bergeri* chromoblastomycosis has been reported in the US, also in an immunosuppressed man without endemic travel, in whom cure was likewise achieved surgically.^7^ *E bergeri* occupies hydrocarbon-rich niches, including creosote-treated wood and guano-rich soil.^8^ It has not been reported from the US environment, although it is recovered from US clinical specimens.^9^

Onset ranges from weeks to months after inoculation. Lesions begin as an erythematous macule or papule that may evolve into verrucous, plaque, tumorous, cicatricial, or nodular forms, sometimes with superficial “cayenne pepper” black dots, satellite lesions, or local extension.^3^^,^^5^ In early stages, the main symptom is pruritus.^3^ Rare cases of lymphatic spread have been reported, though dissemination is extremely rare.^3^ In immunosuppressed hosts, melanized fungi more often cause phaeohyphomycosis, which may disseminate.^3^ *E bergeri,* however, is rarely systemic.^9^ Although slow-growing, if untreated, the disease may progress, resulting in fibrotic changes, lymphedema, or very rarely, squamous cell carcinoma in chronically affected tissue.^3^^,^^5^

To confirm a diagnosis, there must be direct visualization of muriform (sclerotic) cells, often described as Medlar bodies or “copper pennies.”^3^ They contain thick walls with pigmented fungal elements crossed by septa, resembling a brick wall.^5^^,^^10^ PCR can identify the species as in this case.

Diagnosis is frequently delayed because presentations vary and may mimic conditions such as psoriasis, sporotrichosis, leishmaniasis, and other cutaneous infectious processes.^10^ Clinical suspicion may be low in non-endemic regions, particularly in immunocompromised patients, underscoring the importance of maintaining a broad differential.

There is no robust evidence to guide treatment of chromoblastomycosis, and the approach depends on disease severity.^3^ Small lesions (<5 cm) constitute mild disease and can be treated with excision alone or in combination with systemic antifungals.^10^ If patients present later in their course with moderate to severe disease, when larger lesion size and poorly defined margins make excision difficult, management is with systemic therapy alone, most commonly itraconazole or terbinafine.^10^ The length of treatment varies greatly, depending on clinical response. In refractory disease, systemic antifungals may be combined to maximize outcomes.^3^ Other physical removal methods include cryo-, laser-, heat-, and photodynamic therapies; topical imiquimod has been used as an immunomodulatory adjunct to systemic antifungals.^3^

In this patient, initial management was with itraconazole followed by surgical excision. However, due to significant side effects, itraconazole was discontinued. The small size of the lesion suggests excision alone may be sufficient, and he will be closely surveilled by his multidisciplinary care team.

This case highlights the importance of maintaining clinical suspicion for chromoblastomycosis in immunocompromised patients, even in non-endemic regions. Variable clinical morphology may delay diagnosis, particularly when lesions mimic more common processes. Early biopsy is essential for timely diagnosis, which allows for appropriate management and may reduce the risk of disease progression and long-term complications. Awareness of chromoblastomycosis and its varied clinical presentations, especially in immunocompromised patients, is essential for dermatologists practicing in any setting.

### Declaration of generative AI and AI-assisted technologies in the writing process

None.

## Conflicts of interest

None disclosed.
